# The Effect of Omega-3 Fatty Acids on Rheumatoid Arthritis

**DOI:** 10.31138/mjr.31.2.190

**Published:** 2020-06-30

**Authors:** Ifigenia Kostoglou-Athanassiou, Lambros Athanassiou, Panagiotis Athanassiou

**Affiliations:** 1 Department of Endocrinology, Asclepeion Hospital, Voula, Athens, Greece; 2 First Department of Medicine, Asclepeion Hospital, Voula, Athens, Greece; 3 Department of Rheumatology, St. Paul’s Hospital, Thessaloniki, Greece

**Keywords:** Omega-3 fatty acids, experimental arthritis, rheumatoid arthritis

## Abstract

Omega-3 fatty acids are unsaturated fatty acids thought to play a role in health and disease. They are known as essential fatty acids, as they cannot be synthesized in mammals. Omega-3 fatty acids have a beneficial effect on the secondary prevention of coronary artery disease and stroke and are essential for the development and function of the nervous system and the retina in man. Omega-3 fatty acids are thought to have immunomodulatory properties as they act as precursors to lipid mediators of inflammation which may limit or modulate the inflammatory response. Omega-3 fatty acids seem to prevent or attenuate experimental arthritis. They may have a beneficial effect in the treatment of rheumatoid arthritis. Clinical studies have shown that omega-3 fatty acids may have a modulatory effect on disease activity, namely on the number of swollen and tender joints. It appears that omega-3 fatty acids may modulate disease activity in rheumatoid arthritis.

## INTRODUCTION

Omega-3 fatty acids are known for their multiple effects on health and disease. Omega-3 fatty acids have been shown to protect against cardiovascular disease,^1^ playing a role in the secondary prevention of myocardial infarction,^2^ having an anti-arrhythmic action^3^ and reducing inflammation.^4,5^ Omega-3 fatty acids play a role in the treatment of inflammatory bowel disease, and, if taken, may have a beneficial effect.^6^ Omega-3 fatty acids are tested for their therapeutic potential in multiple sclerosis.^7–9^ Omega-3 fatty acids have been shown to have immunomodulatory properties.^10,11^ In this review, the effect of omega-3 fatty acids on rheumatoid arthritis (RA) will be discussed.

Fatty acids are saturated, monounsaturated and polyunsaturated.^12^ The main physical difference between them is that saturated fatty acids are solid at room temperature, while unsaturated fatty acids are liquid. Two types of polyunsaturated fatty acids exist: omega-6 polyunsaturated fatty acids, and omega-3 polyunsaturated fatty acids. Omega-6 fatty acids are available mainly from vegetable oils. Three types of omega-3 fatty acids exist: linolenic acid available from vegetable oils, and eicosapentaenoic acid and docosahexaenoic acid, which must be obtained from marine sources. Linoleic acid, a precursor of the omega-6 series of fatty acids, and α linolenic acid, a precursor of the omega-3 series of fatty acids, are considered essential fatty acids, as they cannot be synthesized in mammals. The names of the fatty acids come from the number of double unsaturated bonds between carbon atoms in the fatty acid chain. Omega-3 fatty acids have their first double bond in the third carbon molecule from the CH_3_ methyl or n or ω end of the molecule, whereas omega-6 fatty acids have their first double bond at the sixth carbon molecule from the CH_3_ methyl or n or ω end of the fatty acid molecule.^13^ Omega-6 linoleic acid can be desaturated in certain plants to form α linolenic acid. Linoleic acid is mainly converted into arachidonic acid, whereas α linolenic acid is elongated and desaturated to form eicosapentaenoic acid and docosahexaenoic acid. Western diet is characterized by the presence of significantly more omega-6 than omega-3 fatty acids the ratio of omega-6/omega-3 reaching as high as 20–30.^14,15^

## MECHANISM OF ACTION

Omega-3 fatty acids have anti-inflammatory action. Eicosanoids are synthesized from omega-6 and omega-3 fatty acids. Arachidonic acid and eicosapentaenoic acid compete for the cyclo-oxygenase and lipoxygenase enzymes for conversion into eicosanoids. Those derived from arachidonic acid are pro-inflammatory and pro-aggregatory, whereas those derived from omega-3 fatty acids are anti-inflammatory and inhibit platelet aggregation. The beneficial effects of omega-3 fatty acids are mediated by themselves as well as their metabolites, namely resolvins, protectins and maresins. The most studied omega-3 metabolites are resolvins, which are classified into two classes. Class D resolvins are products of docosahexaenoic acid and class E resolvins are products of eicosapentaenoic acid.^16–18^ These metabolites of omega-3 fatty acids compete with those of omega-6 to promote the resolution of the inflammatory cycle.^19,20^ They are thought to play a significant role in the attenuation of inflammation and regulation of autoimmunity.^19,20^

Omega-3 fatty acids may modulate proinflammatory cytokine secretion. In a study involving fish oil consumption by RA patients, the level of plasma interleukin-1β (IL-1β) decreased after fish oil consumption.^21^ In a clinical study in healthy volunteers, fish oil supplementation induced reduction of tumour necrosis factor-alpha (TNF-α), IL-1β, and interleukin-6 (IL-6) by endotoxin-stimulated monocytes cells.^22^ In studies with fish oil supplementation, it was observed that docosahexaenoic acid and eicosapentaenoic acid reduce the population CD4+ T cells which produce interferon-γ (IFN-γ) and interleukin-17 (IL-17).^23^ Studies in cultured cells have shown that eicosapentaenoic acid and docosahexaenoic acid inhibit the production of the well-known pro-inflammatory cytokines, namely, TNF-α, IL-1β and IL-6.^24–27^ In a transgenic strain of mice, the fat-1, in which endogenous production of omega-3 fatty acids is possible, inflammatory metabolites are markedly reduced.^28,29^ The suppression of inflammatory cytokines by omega-3 fatty acids may be a contributing factor to the amelioration of clinical signs and symptoms in RA. In other studies, eicosapentaenoic acid and docosahexaenoic acid inhibit the proliferation of human T cells in culture and the production of IL-2.^30,31^ Dietary omega-3 fatty acids have been shown to correct the imbalance in Th1 and Th2 ratios in RA and experimental autoimmune encephalitis.^32,33^ It appears, that proliferation and differentiation of T cells may be regulated by omega-3 fatty acids. In studies in vitro, the expression of major histocompatibility complex (MHC) II and antigen presentation via MHC II may be reduced following exposure to omega-3 fatty acids.^34,35^ These in vitro results have been confirmed in mice and in humans.

**Figure 1.**
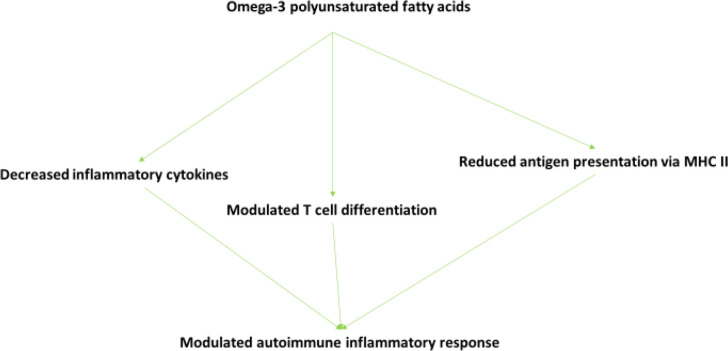
Mode of action of omega-3 polyunsaturated fatty acids on the autoimmune inflammatory response (MHC -major histocompatibility complex).

The above-mentioned data show that omega 3-fatty acids may decrease inflammatory cytokines, modulate T-cell differentiation and reduce antigen presentation via MHC II thus modulating the inflammatory autoimmune response (*Figure 1*).

## EXPERIMENTAL FINDINGS

**Figure 2.**
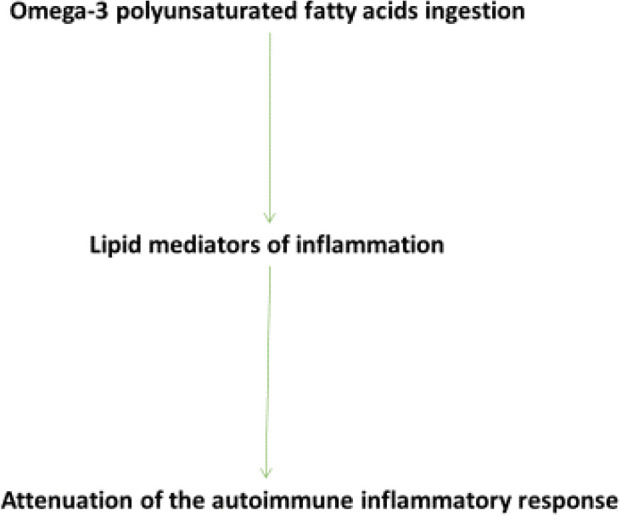
The effect of dietary omega-3 polyunsaturated fatty acids on the autoimmune inflammatory response.

Studies performed in animal models have shown a protective effect of omega-3 fatty acids against experimentally induced arthritis. Fish-oil feeding in mice delayed the onset and reduced the incidence and severity of type II collagen-induced arthritis compared with the vegetable oil-fed group.^36^ In the DBA/1 mouse strain, which is susceptible to the development of collagen-induced arthritis, daily intake of marine omega-3 polyunsaturated fatty acids in the form of phospholipids delayed the onset of arthritis, decreased the severity, reduced paw swelling, and knee joint pathology in collagen-induced arthritis.^37^ In a rat model, eicosapentaenoic acid and docosahexaenoic acid have been shown to suppress Streptococcal-induced arthritis.^38^ In this model, eicosapentaenoic acid appeared to be more effective than docosahexaenoic acid. Endogenous production of omega-3 fatty acids in the fat-1 transgenic mice drastically attenuated arthritis as well as local and systemic levels of inflammatory cytokines following the establishment of RA, whereas the wild type control mice developed overt arthritis.^39^ Docosahexaenoic acid has been shown to ameliorate experimentally induced autoimmune encephalitis in mice.^40^ It appears that dietary-induced changes of tissue levels of polyunsaturated fatty acids modify inflammatory reactions through changes in the synthesis of lipid mediators of inflammation (*Figure 2*).^38^

## CLINICAL FINDINGS

In an early study published in the Lancet in 1985, Kremer et al.^41^ investigated the effect of diet manipulation in RA patients. In a 12-week, prospective, double-blind controlled study, 17 patients received an experimental diet high in polyunsaturated and low in saturated fat, with a daily supplement of 1.8 g eicosapentaenoic acid, while 20 patients received a control diet with a lower polyunsaturated:saturated fat ratio and a placebo supplement. The experimental group at 12 weeks had lower levels of morning stiffness and lower number of tender joints. During follow-up and 1–2 months after stopping the diet, the experimental group had deteriorated significantly. In a subsequent study, Kremer et al.^42^ investigated the effect of omega-3 fatty acids on RA patients. In a 24-week, prospective, randomized, double-blind study involving 49 patients with RA, they studied the effect of high dose fish oil (dietary supplements with 54 mg/kg eicosapentaenoic acid and 36 mg/kg docosahexaenoic acid), low dose fish oil (27 mg/kg eicosapentaenoic acid and 18 mg/kg docosahexaenoic acid) and olive oil on the disease. They observed significant decrease in the number of tender and swollen joints in both groups with omega-3 fish oil supplements, the effect observed earlier in the high dose group. Cytokine production by immune cells also decreased. Namely, neutrophil leukotriene B4 production and macrophage interleukin-1 production decreased significantly after fish oil supplementation. Veselinovic et al.^43^ investigated the effect of omega-3 fatty acid supplementation in 60 patients with RA in a 12-week prospective, randomized trial. Patients received omega-3 fatty acids, omega-3 fatty acids plus evening primrose oil or no supplement. They observed a significant decrease in DAS28, number of tender joints and VAS score in both experimental groups. In a study performed in Sweden involving 737 patients with early RA, omega-3 fatty acid intake was associated with a good response to treatment according to the EULAR criteria.^44^ In a prospective, controlled, double-blind trial performed in Iran involving 61 RA patients, omega-3 fatty acids were administered to the patients along with their standard treatment.^11^ The patients were assessed and significant improvement was noted in the patient’s global evaluation, and in the physician’s assessment of disease in those taking omega-3. The proportions of patients who improved and of those who were able to reduce their concomitant analgesic medication were significantly greater with omega-3 consumption, while no weight change was observed. In a randomized, controlled, double-blind study performed in Austria involving 23 patients with active RA, patients received an infusion of fish oil emulsion for 14 days followed by oral treatment with fish oil capsules.^45^ Swollen joint count was significantly lower in the omega-3 fatty acid group after 1 and 2 weeks of infusion. Tender joint count tended also to be lower in the omega-3 fatty acid group after 1 and 2 weeks of infusion. Both swollen and tender joint counts were significantly lower in the omega-3 fatty acid group on oral treatment compared to the placebo group. In a food frequency study performed in Norway involving 78 RA patients, Beyer et al.^46^ found that seafood intake according to nutritional recommendations was related to better disease outcome. In a study performed in the Netherlands, van der Tempel et al.^47^ investigated the effect of fish oil supplementation on RA and they observed a decrease in morning stiffness and joint swelling index. In a study involving 32 patients with RA, fish oil was administered and plasma interleukin-1β levels decreased.^21^ Lee et al.^48^ performed a meta-analysis on the effect of omega-3 fatty acids on clinical outcomes in RA. Their meta-analysis included 10 randomized controlled trials involving 187 RA patients and 183 placebo-treated RA control subjects. They showed that omega-3 fatty acid consumption reduced NSAIDs consumption without between-study heterogeneity. Tender joint count, swollen joint count, morning stiffness and physical function tended to improve more in patients receiving omega-3 fatty acids, but the effect did not reach statistical significance. Thus, it appears that omega-3 fatty acids may have an anti-inflammatory action and may decrease disease activity in RA.^49^ In the era of precision medicine,^50^ precision nutrition^51^ is a new concept which may modulate the autoimmune response^52^ and may aid in the management of RA.

## CONCLUSION

Omega-3 fatty acids are polyunsaturated fatty acids which have an impact in health and disease. They act as precursors to lipid mediators of inflammation and may attenuate and modulate the autoimmune inflammatory response. They have been shown to ameliorate or prevent experimental arthritis and may decrease disease activity in rheumatoid arthritis.
